# *Notes from the Field*: Meningeal and Pulmonary Tuberculosis on a Commercial Fishing Vessel — Hawaii, 2017

**DOI:** 10.15585/mmwr.mm6824a5

**Published:** 2019-06-21

**Authors:** Erin K. Imada, Emily K. Roberson, Neela D. Goswami, Richard J. Brostrom, Kathleen Moser, Kara Tardivel

**Affiliations:** ^1^Division of Global Migration and Quarantine, National Center for Emerging and Zoonotic Infectious Diseases, CDC; ^2^Hawaii Pacific University, Honolulu, Hawaii; ^3^Division of Tuberculosis Elimination, National Center for HIV/AIDS, Viral Hepatitis, STD, and TB Prevention, CDC; ^4^Hawaii Tuberculosis Control Program, Hawaii Department of Health.

In December 2016, U.S. Customs and Border Protection notified the CDC Honolulu Quarantine Station of a crewman on a commercial fishing vessel who was hospitalized with suspected tuberculosis (TB); the crewman, in his mid-30s, was unconsciousness, intubated, and dependent upon mechanical ventilation to maintain his respiratory status. He was a native of a high TB-burden country (one with TB incidence exceeding 10 cases per 100,000 population per year)* in the Pacific region. Nine days earlier, he had been hospitalized in Hawaii following a 1-month history of headache, fever, night sweats, chills, fatigue, weight loss, breathing difficulties, and cough and recent onset of abdominal pain, vomiting, dizziness, and blurred vision. Brain computerized tomography (CT) and magnetic resonance imaging scans showed lesions in the left basal ganglia and left temporal lobe; chest CT showed multiple bilateral lung opacities with central cavitation. Pathology results from a lung biopsy demonstrated acid-fast bacilli with molecular and culture tests positive for *Mycobacterium tuberculosis* complex, susceptible to all first-line drugs. Cerebrospinal fluid demonstrated low glucose (23 mg/dL), elevated protein (247 mg/dL), and elevated white blood cell count (298 cells/uL) with a relative lymphocytic predominance (50%), consistent with TB meningitis. Testing for human immunodeficiency virus infection was negative, and the patient had no medical comorbidities. The Hawaii Department of Health (Hawaii DOH) was contacted to assist with the investigation.

Upon confirmation of infectious TB, U.S. Customs and Border Protection, accompanied by the Hawaii Harbor Police called the vessel to port for contact interviews and TB screening by the CDC Honolulu Quarantine Station and Hawaii DOH. The vessel’s crew members included the captain, who was a U.S. citizen, and five crew members from the same country as the patient. A fisheries observer with the National Oceanic and Atmospheric Administration, who was a U.S. citizen, was identified as an additional contact. All seven contacts were evaluated by Hawaii DOH; none reported symptoms or signs of active TB. Four crew members had positive tuberculin skin test readings. These four persons were evaluated by Hawaii DOH and had normal chest x-rays, indicating that they had latent TB infection (LTBI). All four were offered LTBI treatment by Hawaii DOH, but all declined. The patient received standard four-drug treatment while hospitalized, regained consciousness and survived respirator-weaning but remained neurologically incapacitated. He completed 12 months of TB treatment. After 426 days, he was repatriated to his country of origin. 

Meningeal TB is the most severe form of TB and is associated with high morbidity (5%–24%) (e.g., stroke, seizure, hydrocephalus, vision impairment, and hearing impairment) and mortality (10%–20%), even in high-resource countries (*1*). Infection of the central nervous system occurs by hematogenous dissemination of *Mycobacterium tuberculosis* from the lungs (*2*). Neurologic complications are most common when diagnosis and treatment are delayed (*2*,*3*).

This case highlights the clinical importance of considering TB in persons from high TB-burden countries. Meningeal TB should be suspected in patients who have signs and symptoms of TB disease (e.g., fever, chills, night sweats) as well as neurologic disease (e.g., headache, blurred vision, dizziness) without a plausible alternative diagnosis by history, physical exam, and basic laboratory or radiographic studies. Once extrapulmonary TB is confirmed, an evaluation for pulmonary disease should proceed (*4*). This patient’s delay of >1 month before being evaluated by a health care provider and his resultant neurologic incapacitation highlight the importance of early diagnosis and the challenges associated with accessing health care among mobile populations. Commercial, noncruise maritime crew members might be particularly vulnerable because of their remote locations, lack of onboard medical resources, and possible inadmissibility to the nearest port of entry. Crew members on commercial vessels are also at high risk for infection if exposed to communicable disease on board because of their close living and working conditions. 

A number of factors affect initiation of LTBI therapy in mobile populations, including provider communication about treatment for LTBI versus active disease, barriers to access LTBI therapy, duration of treatment, financial and medical risk of medications, patient willingness to accept therapy, and provider willingness to prescribe treatment given the risk for loss to follow-up. Approximately 4%–6% of persons with untreated LTBI will develop infectious TB (*4*). Although the four contacts with LTBI declined treatment, this contact investigation demonstrates the feasibility of successful evaluation and management of TB close contacts even in challenging settings.

## Footnotes

* Countries with the highest number of incident TB cases and that account for the highest global percentage of new TB cases. https://www.tbfacts.org/countries-tb/.
